# Functional characteristics of cancer stem cells and their role in drug resistance of prostate cancer

**DOI:** 10.3892/ijo.2014.2529

**Published:** 2014-06-27

**Authors:** VIVIANA CASTILLO, RODRIGO VALENZUELA, CHRISTIAN HUIDOBRO, HECTOR R. CONTRERAS, ENRIQUE A. CASTELLON

**Affiliations:** 1 Laboratory of Molecular and Cellular Andrology, Physiology and Biophysics Program, Institute of Biomedical Sciences, Faculty of Medicine, University of Chile, Santiago 8380453, Chile; 2 Urology Service, Clinical Hospital, University of Chile, Santiago 8380453, Chile

**Keywords:** cancer stem cells, prostate cancer, multidrug resistance

## Abstract

Cancer stem cells (CSCs) have the ability to self-renew and differentiate to give rise to heterogeneous phenotype of the tumor cells. It is believed that these cells are involved in metastasis, recurrence and therapy resistance in various cancers. CSCs have been identified in prostate cancer (PCa), one of the most diagnosed malignancies in men over the world, for which chemotherapy resistance is a major problem in the treatment of castration-resistant advanced stages. Molecular signatures, gene expression and functional features have been reported for PCa CSCs. Most data come from cell lines which may not represent the actual tumor. In the present work, a CSCs enriched population obtained from PCa explants was functionally characterized and analyzed for drug resistance. Tumorsphere cultures positive for ABCG2 transporter, CD133, CD44, cytokeratins 5 and 18 (CK5 and CK18) and negatives for androgen receptor (AR) and prostate-specific antigen (PSA) showed higher clonogenic capacity, holoclone-forming ability, colony-forming capacity in soft agar and lower proliferative and apoptotic rate than control adherent cell cultures. Furthermore, exposing tumorsphere cultures to ABCG2 substrate drugs resulted in a high survival rate compared with control PCa cells. This high drug resistance was decreased using a selective inhibitor of ABCG2. According to these results, tumorspheres from PCa explants showed a functional stem phenotype and a marked drug resistance, probably mediated by high expression of the ABCG2 transporter, which might be considered as a suitable therapeutic target for CSCs.

## Introduction

Cancer stem cells (CSCs) have been identified in various cancers and are thought to be involved in metastasis, recurrence and radio/chemotherapy resistance (1–5). One of the main properties used to isolate CSCs is their capacity to form spheres in non-adherent culture conditions (6). Recently CSCs have been identified in prostate cancer (PCa) (7,8), one of the most diagnosed male malignancies worldwide (9). Several PCa CSCs features such as molecular signatures, gene expression profiles, functional characteristics and metastatic potential have been published (10,11). Most data come from CSCs produced from PCa cell lines, mainly from metastasis, and animal models which are the main bias for the clinical projection of the results. These studies have identified several molecular markers for CSCs such as cluster of differentiation 133, 44, 40 (CD133, CD44, CD40) and α2β1 integrin (12,13). Furthermore, the capacity to exclude Hoechst 33342 staining has been used to separate CSCs (side population) (14,15). ABCG2 transporter is responsible for the exclusion of this dye and has been found overexpressed in several CSCs, including PCa (16). Recently, we have studied several stem markers in biopsy archives of PCa tumors of different Gleason grades, lymph node and bone metastases, and also have obtained an enriched CSCs population from PCa explants (13). These tumorsphere (prostatosphere) cultures showed a molecular signature CD133^+^/CD44^+^/ABCG2^+^/CD24^−^, and these stem markers were found mainly in medium Gleason samples of PCa biopsies (13). On the other hand, PCa is known by its strikingly high intrinsic drug resistance (17–19). In advanced disease, the gold standard is androgen-deprivation therapy (20,21) but in castration-resistant stages, chemotherapy has very limited impact in patient survival (22). Previously, we have extensively studied the molecular mechanisms of multidrug resistance (MDR) in PCa (23,24). A high expression of ABC transporters seems to be involved in this MDR phenotype, since the pharmacological blockage and knocking down of ABC transporters partially sensitize PCa cells to therapeutic drugs (23). PCa CSCs exhibit a high expression of ABCG2 (13), a transporter used by several chemotherapeutic drugs, suggesting that CSCs may be responsible for the increased MDR phenotype of PCa tumors. In the present work, a functional characterization, including drug resistance, of prostatosphere cultures obtained from PCa explants is reported.

## Materials and methods

### Prostatic tissue

The prostatic tissue was obtained from patients undergoing radical prostatectomy for PCa from the Clinical Hospital of the University of Chile. The tissue was received in sterile culture medium and transferred subsequently to the laboratory. All protocols for obtaining these samples were approved by the Committees of Bioethics of the Faculty of Medicine and the Hospital of the University of Chile.

### Cell cultures

PCa cell cultures were obtained from the tumor explants according to our methods described (13). After enzymatic digestion, resulting cell aggregates were washed and seeded in culture plates of 10 cm diameter using DMEM/F-12 culture medium (Gibco, Invitrogen, Carlsbad, CA, USA) including fetal bovine serum 7% (FBS), and supplements as described previously (13), in an atmosphere of 5% CO_2_ at 37°C. These cultures in adherent conditions contained a representative mixed population of tumor cells and were considered as non-CSC controls.

### Tumorsphere (prostatosphere) cultures

After 3 passages of the cell cultures described above, cells were detached, washed and cultured in non-adherent conditions in absence of FBS and with B-27 supplement (Gibco, Invitrogen), as described previously (13). Resulting tumorspheres were maintained until at least 2 weeks with medium change every 3 days. These prostatosphere cultures contained mainly cells with stemness markers and were considered as CSCs.

### Immunocytochemistry

Adherent cells at 60% confluence were washed, trypsinized, collected and centrifuged at 70 g, whereas, the prostatospheres were collected on day 10 of culture, washed and pelleted. Subsequently, both pellets from prostatospheres and adherent cells, were fixed with paraformaldehyde solution (paraformaldehyde 16%, PBS 1 M, sucrose 0.2 M) for 12 h. Afterwards, the pellets were washed and embedded in HistoGel (Thermo Scientific, Waltham, MA, USA) a matrix that preserves cellular integrity and facilitates handling during histological processing. Then, samples were dehydrated in an increasing ethanol concentration, cleared in butanol and embedded in Paraplast Plus. Serial sections (3-μm) were obtained and mounted on silanized slides. Samples from adherent and prostatosphere cultures were deparaffinized in xylene and hydrated in a decreasing concentration of ethanol. Subsequently, antigen retrieval was performed and the preparations were transferred to citrate buffer pH 6.0, exposed for 3 min in a microwave oven and immediately incubated for 30 min in a steamer. Then, the preparations were incubated in H_2_O_2_ 3% to inhibit endogenous peroxidases and then washed in distilled water. Then, sections were incubated for 30 min in normal goat serum to block non-specific binding. Later, samples were incubated at 4°C for 12 h with antibodies to anti-stemness markers, CD44 (Santa Cruz Biotechnology, Inc., Santa Cruz, USA), CD133 (Bioss, Woburn, MA, USA) and cytokeratin 5 (CK5) (Thermo Scientific), and anti-differentiation markers, androgen receptor (AR) (Thermo Scientific), prostatic specific antigen (PSA) (Santa Cruz Biotechnology, Inc.) and cytokeratin 18 (CK18) (Abcam, Cambridge, MA, USA). Then, the samples were washed and incubated for 1 h at room temperature with the corresponding secondary antibody (goat, anti-mouse, KPL, Inc., Gaithersburg, MD, USA). After washing, the slides were incubated for 30 min at room temperature with immuno-peroxidase kit (Vectastain-ABC, Vector Laboratories, Burlingame, CA, USA), washed and incubated for 10 min with 3,3′-diaminobenzidine substrate (DAB) (Sigma, St. Louis, MO, USA) as chromogen, followed by counterstaining with hematoxylin. Subsequently, sections were dehydrated in increasing ethanol concentrations, cleared in xylene and mounted with Entellan (Merck, Germany). For negative control, serial sections were exposed only to the secondary antibody. For immunostaining intensity quantification, H-Score method was used (25).

### Differential cloning capacity

To evaluate the formation of different types of clones (holoclones, meroclones and paraclones), prostatospheres obtained at day 7 of culture and adherent cultures at 60% confluence, were digested with Accutase (StemCell Technologies, USA) at 37°C for 10 min and then disrupted mechanically by pipetting. Resulting cells were cultured at density of 2000 cells/well in plates of 6 cm diameter for 15 days at 37°C in 5% of CO_2_. Subsequently, the colonies were stained and fixed with glutaraldehyde 6% mixed 1:1 with crystal violet 0.5% for 30 min (26). Then, the plates were washed with water and dried at room temperature. For counting, only colonies with >50 cells were considered (26). The morphology of the colonies was evaluated according to a method described previously (27), under a stereoscopic microscope connected to a digital camera Olympus C-4040 DIG CAM Zoom. The results were expressed as the percentage of each clone type formed in both prostatospheres and adherent cell cultures.

### Anchorage-independent growth capacity (soft agar assay)

To assess anchorage-independent colony formation capacity, prostatospheres obtained at day 7 of culture and adherent cultures at 60% confluence, were digested with Accutase at 37°C for 10 min and then disrupted mechanically by pipetting. Resulting cells were resuspended in culture medium mixed 1:1 with agarose 0.3% and plated at density of 1000 cells/well in plates of 6 cm diameter, coated with agar 1%, for 15 days at 37°C in 5% of CO_2_. Subsequently, plates were stained with crystal violet 0.5% and fixed with methanol for 30 min (26). Then, the plates were washed with water and dried at room temperature. For counting, only colonies with diameter >100 μm were considered (28). The results were expressed as the number of colonies formed from each type of cell culture.

### Single colony formation assay

To evaluate the single colony formation capacity, prostatospheres obtained at day 7 of culture and adherent cultures at 60% confluence, were digested with Accutase at 37°C for 10 min and then disrupted mechanically by pipetting. Resulting cells were seeded at a density of 1 cell/well in 96-well culture plates coated with agarose 1%, for 15 days at 37°C in 5% of CO_2_. Subsequently, the spheres formed were counted using a phase contrast microscope (29).

### Evaluation of cell proliferation

Cell proliferation rates of prostatospheres and adherent cultures were evaluated by immunocytochemistry, following the protocol and conditions described above, using antibodies against the proliferation markers Ki67 (Dako/Agilent Technologies, Santa Clara, CA, USA), PCNA (Novocastra, UK) and BrdU (Zymed, Life Technologies USA).

### Evaluation of apoptosis

Apoptosis was evaluated using the TUNEL assay (*In situ* cell death detection kit Rhodamine; Roche Diagnostics GmbH, Mannheim, Germany). Both prostatospheres and adherent cells were processed as for immunocytochemistry. The preparations obtained were subjected to TUNEL-Rhodamine technique following the manufacturer’s instructions. Slides were blocked with BSA 3%, and exposed to TUNEL assay at 37°C for 1 h in a moist chamber in darkness. For negative control, serial sections were exposed only to the staining solution. As a positive control, cells exposed to daunorubicin 8 μM (Sigma) were used. The preparations obtained were analyzed in a confocal laser scanning microscope, Nikon C1 Plus model.

### Drug resistance assay

Both adherent cells and prostatospheres were cultured for 7 days, in their specific conditions, in 48-well plates at 37°C in 5% of CO_2_. Following this, the media were changed to include topotecan (Sigma) or daunorubicin (Sigma), drugs that are substrates for ABCG2 transporter, at different concentrations for 48 h. Afterwards, culture media containing the drugs were removed and replaced with 100 μl of MTT (dimethyl-thiazol-diphenyl tetrazolium) solution (Sigma). The incubation was performed for 2 h at 37°C in darkness. After the incubation, the MTT solution was removed and replaced by DMSO and plates incubated with stirring at room temperature. Then, each plate was analyzed in a micro-plate reader at 570 nm (BioTek Instruments, Inc., Winooski, VT, USA). The results were expressed as the percentage of survival respect to the control cells incubated without drugs, which were considered as 100% survival. Furthermore, in parallel experiments, the ABCG2 inhibitor fumitremorgin C (Sigma) was used alone or in combination with both drugs. For topotecan and daunorubicin, dose-response curves for each culture type (adherent and prostatospheres) were carried out. The corresponding half maximal effective concentrations (EC50) for both drugs were determined by analyzing the resulting dose-response curve using GraphPad Prism 6.0 software.

### Statistical analysis

The statistical evaluation of the results was performed using unpaired two-tailed Student’s t-test or ANOVA followed by Bonferroni post test. Statistic significance was considered for p<0.05. Results were expressed as means ± SE.

## Results

### Expression of stemness and differentiation markers in prostatospheres

**Figure 1:**
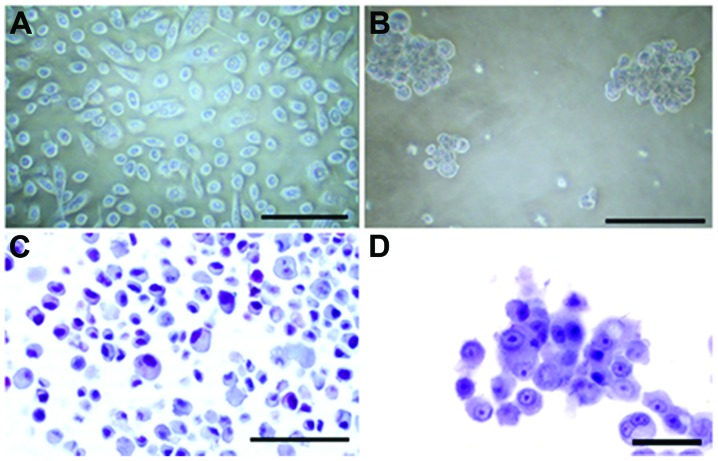
Culture types. Adherent control (A and C) and tumorsphere cell cultures (B and D). (A and B) Phase-contrast microscopy images. (C and D) Histological haematoxylin images. Scale bars: (A) 150 μm; (B) 100 μm; (C) 80 μm; (D) 30 μm.

**Figure 2:**
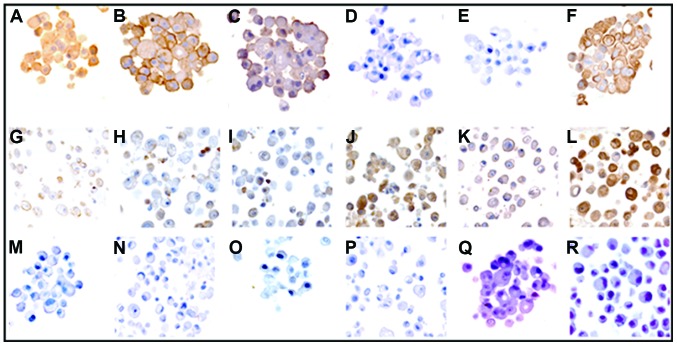
Expression of stemness and differentiation markers. (A–F) Tumorspheres. (G–L) Adherent control cells. (A and G) Cluster of differentiation 133 (CD133); (B and H) Cluster of differentiation 44 (CD44); (C and I) Cytokeratin 5 (CK5); (D and J) Androgen receptor (AR); (E and K) Prostate specific antigen (PSA); (F and L) Cytokeratin 18 (CK18). (M–R) Corresponding negative controls. Magnification, ×400.

**Figure 3:**
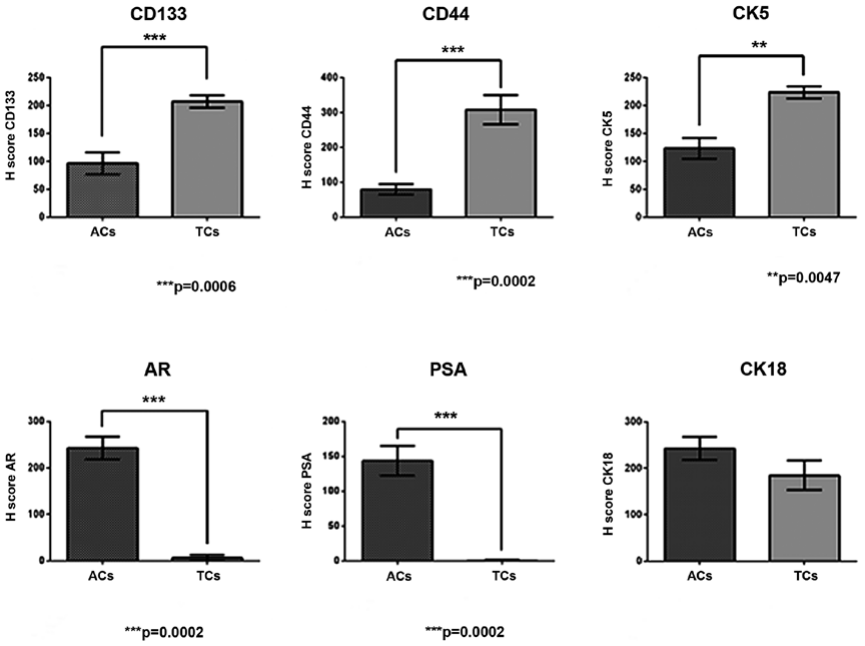
H-Scores for stemness and differentiation markers in tumorspheres and adherent control cultures. CD133, cluster of differentiation 133; CD44, cluster of differentiation 44; CK5, cytokeratin 5; AR, androgen receptor; PSA, prostate specific antigen; CD18, cytokeratin 18. ACs. Adherent cultures TCs, tumorsphere cultures. Data are expressed as means ± SE, n=8.

Stemness markers CD133, CD44 and CK5, and differentiation markers AR, PSA and CK18 were analyzed in prostatospheres and adherent control cell cultures, by immunocytochemistry. Morphology of both types of cultures is shown in Fig. 1. Prostatospheres are highly positive for CD133, CD44, CK5 and CK18 (Fig. 2A–C and F, respectively). However, spheres were almost negative for AR and PSA (Fig. 2D and E, respectively). Adherent control cell cultures exhibited very weak staining for stemness markers (Fig. 2G–I), and strongly positive staining for differentiation markers (Fig. 2J–L). Haematoxylin and eosin controls are also shown (Fig. 2M–R). Immunostaining quantification of each marker for prostatospheres and control cultures are shown in Fig. 3.

### Differential cloning capacity

**Figure 4:**
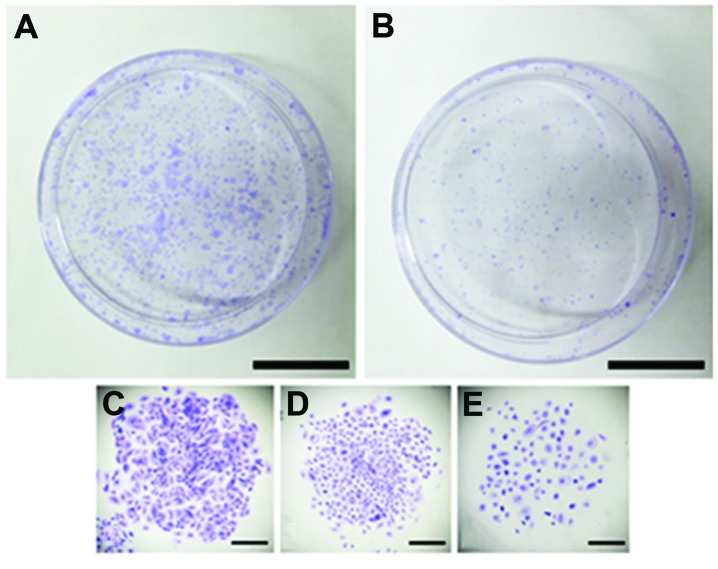

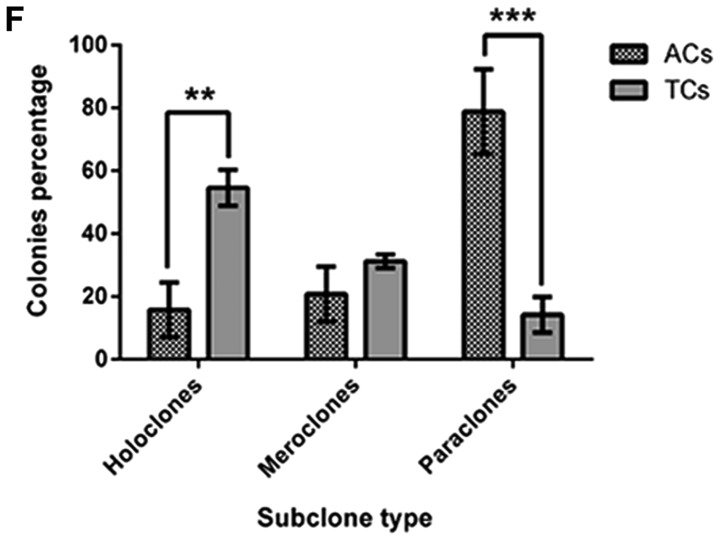
Differential cloning capacity of cells from different cultures. (A) Total colonies formed by cells derived from tumorsphere cultures; (B) Total colonies formed by cells derived from adherent control cultures; (C) Holoclones; (D) Meroclones; (E) Paraclones. Scale bars: (A and B), 20 mm; (C–E), 250 μm; (F), comparison between percentage of sub-clone types from tumorspheres (TCs) and adherent control cultures (ACs). Data are expressed as means ± SE. ^**^p=0.008, ^***^p<0.0001, n=5.

The ability to form holoclones (compact colonies), meroclones (loose colonies) and paraclones (dispersed colonies) was evaluated in prostatospheres and adherent cultures (Fig. 4A and B). Only clones formed by at least 50 cells were considered. The formation of the three types of clones was observed in both cultures (Fig. 4C–E). PCa tumorspheres formed a larger percentage of holoclones than control adherent cultures (Fig. 4F), whereas, the cells from adherent cultures formed a high percentage of paraclones compared to cells from spheres cultures (Fig. 4F). Meroclones were observed in both types of cultures without significant difference (Fig. 4F).

### Anchorage-independent growth capacity

**Figure 5:**
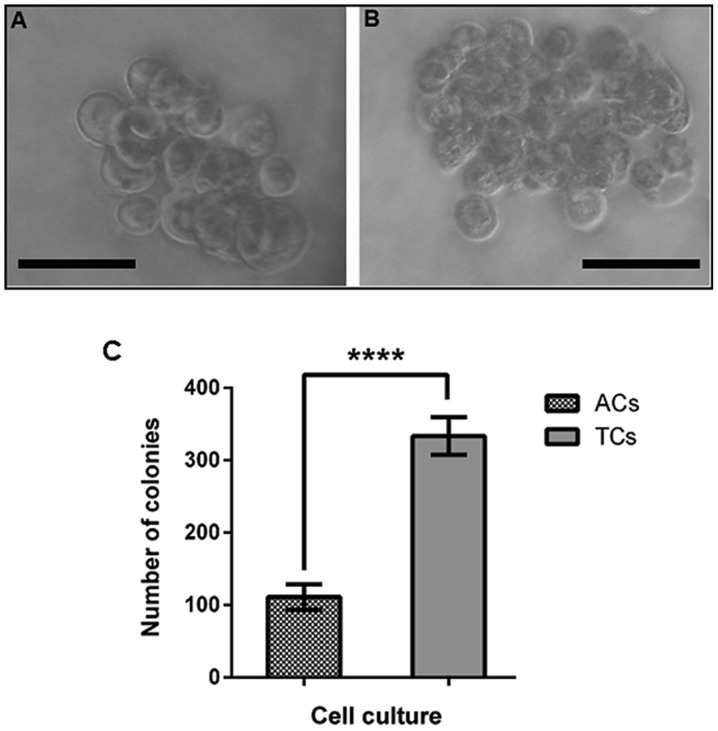
Anchorage-independent growth capacity in soft agar of cells from different cultures. (A) Representative colony from cell adherent cultures; (B) Representative colony from tumorsphere cultures. Scale bars, 100 μm; (C) Comparison between the colony number formed in soft agar of cells from tumorspheres (TCs) and adherent cultures (ACs). Data are expressed as means ± SE. ^****^p=0.0006, n=7.

The ability to form colonies in soft agar, in an anchorage-independent manner, was assessed in prostatospheres and adherent control cells. Only colonies with diameter >100 μm were considered. Spheroid-derived colonies reached a diameter larger than the colonies formed from adherent cultures (Fig. 5A and B). In addition, it was observed that cells derived from tumorspheres formed a larger number of colonies than cells from adherent cell cultures (Fig. 5C).

### Single colony formation ability

**Figure 6:**
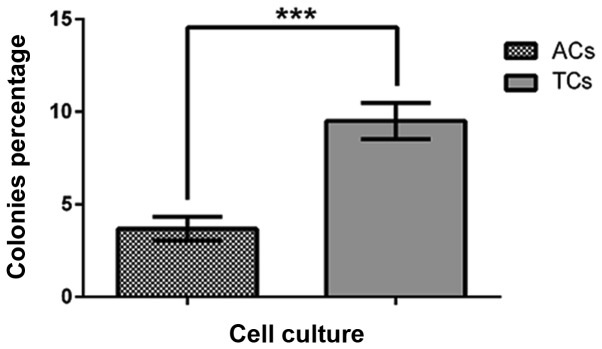
Single colony formation ability. Comparison of the ability to form a colony from a single cell of tumorsphere cultures (TCs) and adherent control culture (ACs). Cells were seeded at a density of 1 cell/well in 96-well coated with agarose 1%. Data are expressed as means ± SE. ^***^p=0.0006, n=7.

The ability to form colonies from a single cell, an assay often used to assess self-renewal potential, was evaluated in prostatospheres and adherent control cultures. Single cells from PCa tumorspheres showed a significantly higher percentage of colonies compared with cell from control adherent cultures (Fig. 6).

### Cell proliferation activity

**Figure 7:**
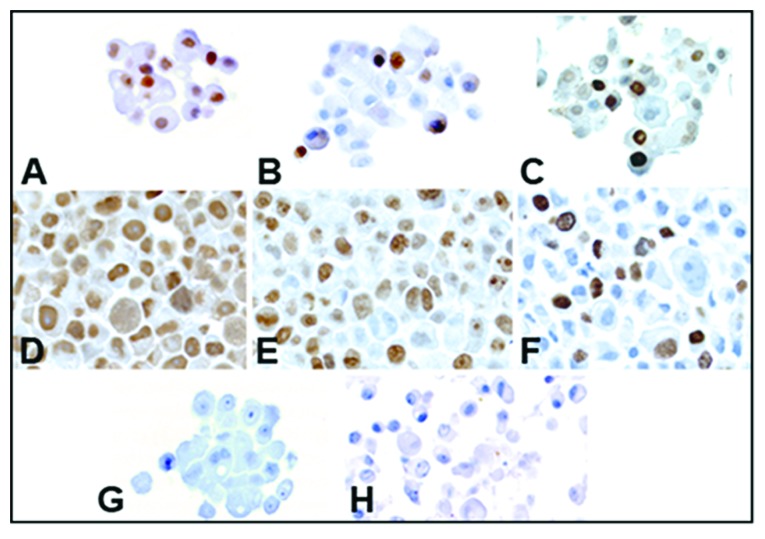

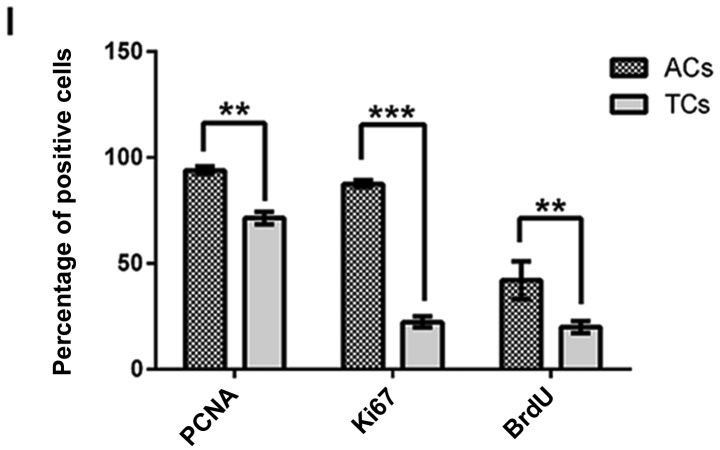
Proliferation rate assessed by Ki67, PCNA and BrdU immunostaining. (A–C) Tumorspheres; (D–F) Adherent cells; (A and D) PCNA; (B and E) Ki67; (C and F) BrdU; (G and H) Corresponding negative controls; (I) Percentage of positive cells for each proliferation marker from tumorsphere cultures (TCs) and adherent control cultures (ACs). Magnification, ×400. Data are expressed as means ± SE. ^**^p=0.0016, ^***^p<0.0001, n=9.

Cell proliferation in prostatospheres and adherent cell cultures was assessed using immunocytochemistry for Ki67, PCNA and BrdU (Fig. 7A–F). Haematoxylin and eosin controls are also shown (Fig. 7G and H). Tumorspheres cultures showed a smaller number of positive cells than adherent cultures for the three proliferation markers (Fig. 7I). Furthermore, the Ki67 marker showed the largest difference between the culture types (Fig. 7I).

### Apoptosis

**Figure 8:**
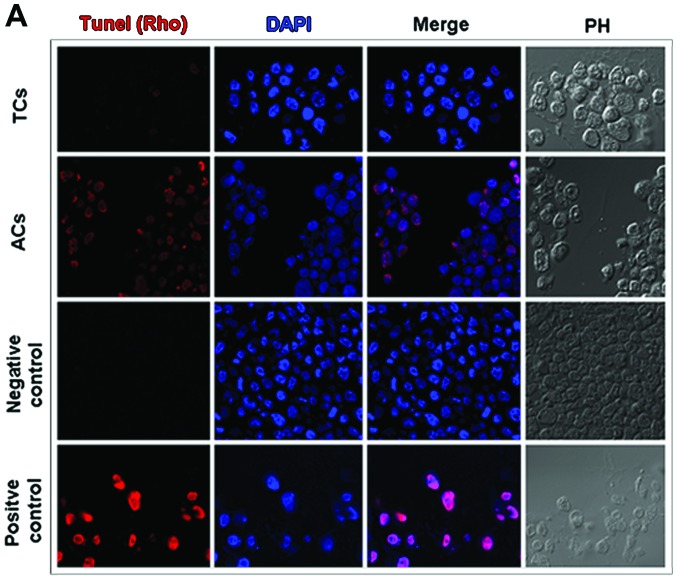

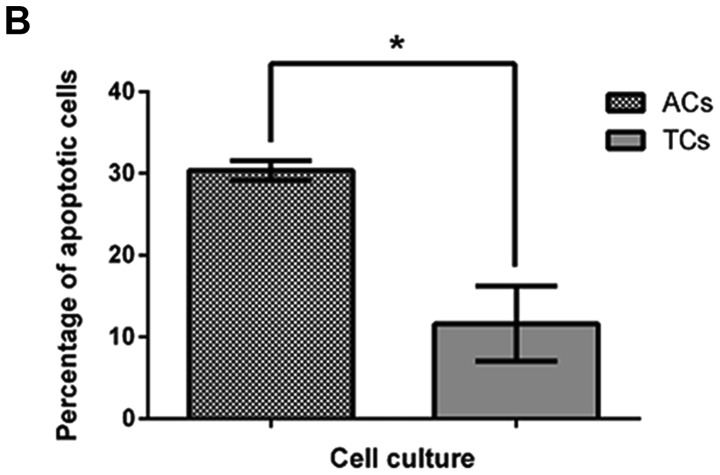
Apoptosis rate assessed by TUNEL-Rhodamine assay. (A) TUNEL assay performed on tumorspheres (TCs) and adherent cultures (ACs). DAPI; merge and phase contrast (PH) images are shown for comparison. Negative (only staining solution) and positive (daunorubicin 8 μM) controls are provided. Magnification, ×400. (B) Percentage of apoptotic cells from tumorspheres (TCs) and adherent (ACs) cultures. Data are expressed as means ± SE. ^*^p=0.043, n=6.

Programmed cell death was assessed, by the TUNEL method, both in prostatospheres and adherent control cultures (Fig. 8A). Adherent cells exposed to daunorubicin 8 μM were used as positive control. Tumorspheres showed significantly smaller number of apoptotic cells than adherent cultures (Fig. 8B).

### Drug resistance and ABCG2 transporter in prostatospheres

**Figure 9:**
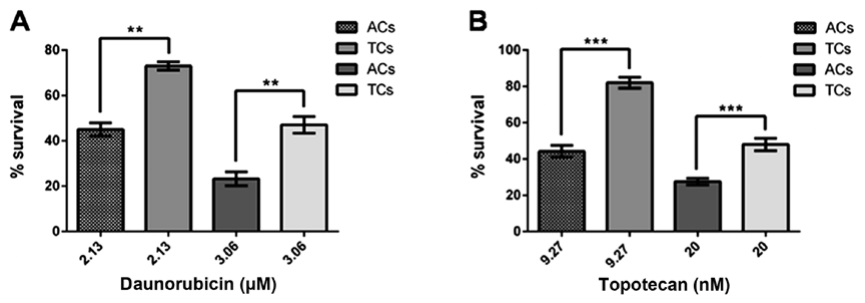
Effect of chemotherapeutic drugs, substrates for ABCG2 transporter, on survival of tumorspheres and adherent cells. (A) Daunorubicin at EC50 for tumorspheres (TCs), 3.06 μM and adherent cells (ACs), 2.13 μM, for 48 h; (B) Topotecan at EC50 for tumorspheres (TCs), 20.0 nM and adherent cells (ACs), 9.27 nM, for 48 h. Cell cultures without treatment were considered as 100% survival. Data are expressed as means ± SE. ^**^p<0.05, ^***^p<0.01, n=6.

**Figure 10:**
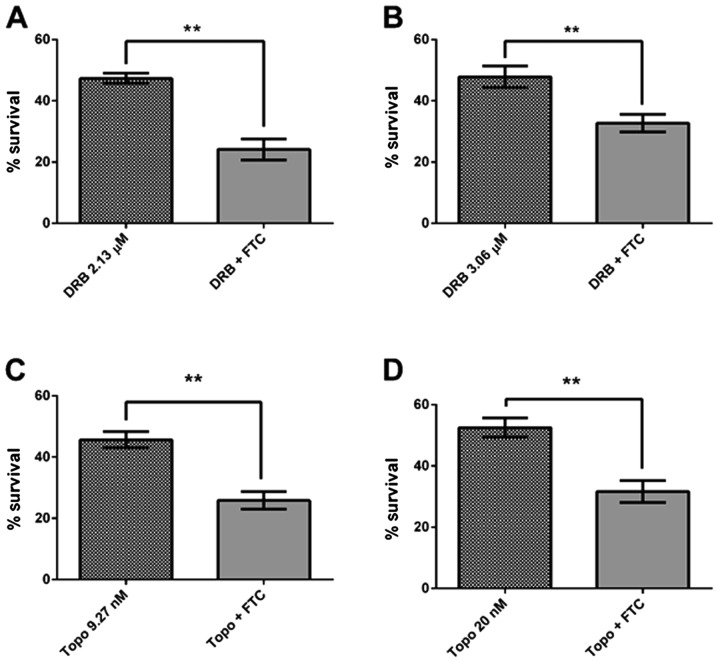
Effect of fumitremorgin C, selective inhibitor of ABCG2 transporter, on daunorubicin and topotecan sensitivity of tumorspheres and adherent cells. (A and C) Adherent cells; (B and D) Tumorspheres. DRB, daunorubicin; Topo, topotecan; FTC, fumitremorgin C 5 μM. All treatments were carried out for 48 h. Cell cultures without treatment were considered as 100% survival. Data are expressed as means ± SE. ^**^p<0.05, n=6.

To assess the cell sensitivity to chemotherapeutic drugs, both prostatospheres and control cultures were treated with topotecan or daunorubicin at their corresponding EC50 previously determined for each drug. Daunorubicin EC50 was 2.13 and 3.06 μM for adherent cells and prostatospheres, respectively. Topotecan EC50 was 9.27 and 20 nM for adherent cells and prostatospheres, respectively. Also, the pharmacological inhibitor of ABCG2 transporter, fumitremorgin C, was used to evaluate the influence of this transporter in the drug resistance phenotype. Cell survival was evaluated using MTT assay. Prostatospheres showed higher cell survival (more resistance) than adherent cells to both drugs used (Fig. 9). In addition, fumitremorgin C 5 μM, a concentration that does not affect cell survival (data not shown), re-sensitized cells, from both types of cultures, to daunorubicin and topotecan when treated with their corresponding EC50 (Fig. 10).

## Discussion

CSCs represent a small cell population within malignant tumors. They have been identified in several cancer types, including PCa, and are thought to be involved in metastasis, relapse and therapy resistance (11,30–32). PCa is a frequent malignancy in men in most countries (9). In advanced castration-resistant stages, chemotherapy is the only therapeutic option. Unfortunately, PCa exhibits a high intrinsic drug resistance so the treatment has little impact on patient survival (18,19). Most studies on PCa CSCs come from cell lines and animal models (8,33,34) limiting the clinical conclusions. Only a few reports obtaining tumorspheres from PCa explants have been published (13,35). The main feature of CSCs is their ability to form spheres when growing in non-adherent conditions. Many tumorspheres have been obtained and characterized from different cancer samples. Most of them exhibit high expression of stemness markers (6,33). Recently, we obtained tumorspheres from PCa explants (13). These prostatospheres show a molecular signature compatible with CSCs. Also, we have found that the same stemness markers observed in CSCs from explants are most expressed in medium Gleason grade biopsies (13). On the other hand, we have extensively studied the multidrug resistance phenomenon affecting PCa (23,24). The involvement of ABC transporter has been clearly demonstrated (23). In our PCa tumorspheres, as in other models, ABCG2 transporter is highly expressed. Indeed, this transporter has been used to isolate CSCs from many tumors (side population). In the present work we confirmed the high expression of stemness markers CD133 and DC44 (13). Also, CK5 and CK18 expression were observed in tumorspheres. This is an interesting finding since CK5 is expressed mainly in prostate basal cells (33) and has been extensively associated with invasiveness and metastasis in breast cancer (36,37). Furthermore, this cytokeratin has also been found in PCa spheres (35), suggesting a contribution of basal cells in CSCs population. The presence of epithelial marker CK18 support the idea that PCa CSCs would come from a divergence of the epithelial mesenchymal transition (EMT) process rather than a malignant transformation of normal prostate stem cell (38). The absence of AR expression suggests that PCa CSCs are the most androgen-resistant cell type within the tumor. Low expression of PSA in prostatospheres may be consequence of EMT. Our results on differential clonogenic capacity (mainly holoclones), anchorage-independent growth and self-renewal properties are absolute congruent with the main features of stem cells published in the literature (33,39,40). Also, low proliferation and apoptotic rate are common characteristic of CSCs. Ki67 protein showed the largest difference between prostatospheres and control cultures. This may be due to the protein being present during all cell cycle phases (41), while PCNA is mainly synthetized in G1 and S phases (42) and BrdU evidence only DNA synthesis (42,43). ABCG2 is one of the most highly expressed ABC transporters in CSCs, including our prostatosphere preparation. Therefore, we have tested the sensitivity of PCa tumorspheres to daunorubicin and topotecan, both ABCG2 substrates and commonly used chemotherapeutic drugs. The corresponding dose-response curves resulted in a higher EC50 for prostatospheres than for control adherent cultures with both drugs (3.06 vs. 2.13 μM for daunorubicin and 20 vs. 9.27 nM for topotecan), showing that CSCs are significant more resistant than other cancer cells to ABCG2 transported drugs. Interestingly, fumitremorgin C, a selective pharmacologic inhibitor of the ABCG2 transporter, re-sensitizes, at least partially, the prostatospheres and adherent cells to both drugs, when used at their corresponding EC50. This result suggests strongly that ABCG2 transporter is involved in drug resistance and may be a suitable therapeutic target for CSCs-selective therapy in PCa, especially in castration-resistant advanced stages.
